# Constitutive production of nitric oxide leads to enhanced drought stress resistance and extensive transcriptional reprogramming in *Arabidopsis*

**DOI:** 10.1093/jxb/eru184

**Published:** 2014-05-27

**Authors:** Haitao Shi, Tiantian Ye, Jian-Kang Zhu, Zhulong Chan

**Affiliations:** ^1^Key Laboratory of Plant Germplasm Enhancement and Specialty Agriculture, Wuhan Botanical Garden, Chinese Academy of Sciences, Wuhan 430074, China; ^2^University of Chinese Academy of Sciences, Beijing, 100039, China; ^3^Shanghai Center for Plant Stress Biology and Institute of Plant Physiology and Ecology, Chinese Academy of Sciences, Shanghai 200032, China; ^4^Department of Horticulture and Landscape Architecture, Purdue University, West Lafayette, IN 47907, USA

**Keywords:** Abscisic acid, drought stress, *in vivo*, neuronal nitric oxide synthase, nitric oxide, physiological, PYL, transcriptomic.

## Abstract

Nitric oxide (NO) is involved in plant responses to many environmental stresses. Transgenic *Arabidopsis* lines that constitutively express rat neuronal NO synthase (nNOS) were described recently. In this study, it is reported that the nNOS transgenic *Arabidopsis* plants displayed high levels of osmolytes and increased antioxidant enzyme activities. Transcriptomic analysis identified 601 or 510 genes that were differentially expressed as a consequence of drought stress or nNOS transformation, respectively. Pathway and gene ontology (GO) term enrichment analyses revealed that genes involved in photosynthesis, redox, stress, and phytohormone and secondary metabolism were greatly affected by the nNOS transgene. Several *CBF* genes and members of zinc finger gene families, which are known to regulate transcription in the stress response, were changed by the nNOS transgene. Genes regulated by both the nNOS transgene and abscisic acid (ABA) treatments were compared and identified, including those for two ABA receptors (*AtPYL4* and *AtPYL5*). Moreover, overexpression of *AtPYL4* and *AtPYL5* enhanced drought resistance, antioxidant enzyme activity, and osmolyte levels. These observations increase our understanding of the role of NO in drought stress response in *Arabidopsis*.

## Summary

Increased endogenous NO content modulates ROS accumulation and related antioxidant enzyme activities, osmolyte levels, and the expression of stress-responsive genes, such as *AtPYL4*/*5*, resulting in enhanced drought resistance in *Arabidopsis*.

## Introduction

As a gaseous diatomic radical, nitric oxide (NO) is an essential endogenous signalling molecule involved in multiple physiological processes in plants, including growth, development, and response to environmental stresses (Shi *et al*., 2012*b*, *c*). Interestingly, NO is rapidly induced by multiple hormonal and environmental stimuli, including abscisic acid (ABA) (Guo *et al.*, 2003), hydrogen peroxide (H_2_O_2_) (Bright *et al.*, 2006), polyamines (Tun *et al.*, 2006; Shi and Chan, 2013, 2014; Shi *et al.*, 2013*a*; Wang *et al.*, 2011), auxin (Kolbert *et al.*, 2007), salicylic acid (SA) (Zottini *et al.*, 2007), brassinosteroids (BRs) (Cui *et al.*, 2011), drought (Fan and Liu, 2012), salt (Zhao *et al.*, 2007; Corpas *et al.*, 2009), cold (Zhao *et al.*, 2009), and heat (Bouchard and Yamasaki, 2008). NO can also act as a secondary messenger in environmental stress signal transduction (Gupta *et al.*, 2011; Gill *et al.*, 2013).

Understanding the complex effects of NO in plants requires a detailed analysis of the physiological and molecular changes. In recent years, transcriptional analyses of plant response to NO have been performed using different techniques (Huang *et al.*, 2002; Polverari *et al.*, 2003; Parani *et al.*, 2004; Grün *et al.*, 2006; Palmieri *et al.*, 2008; Ahlfors *et al.*, 2009; Besson-Bard *et al.*, 2009). These studies have identified thousands of NO-responsive genes, most of which are stress related and serve a variety of functions ranging from plant defence and oxidative stress response to hormonal interplay (Huang *et al.*, 2002; Polverari *et al.*, 2003; Parani *et al.*, 2004; Grün *et al.*, 2006; Ahlfors *et al.*, 2009; Besson-Bard *et al.*, 2009). Further bioinformatics analysis identified several common transcription factor-binding sites (TFBSs) that are enriched in the promoters of these NO-responsive genes, such as WRKY, GBOX, and octopine synthase element-like sequence (OCSE) (Palmieri *et al.*, 2008). However, most of these results were obtained by exogenous application of NO donors such as sodium nitroprusside (SNP), *S*-nitroso-*N*-acetyl-d-penicillamine (SNAP), and nitrosoglutathione (GSNO), NO scavengers such as 2-[4-carboxyphenyl]-4,4,5,5-tetramethylimidazoline-1-oxy-3-oxide (c-PTIO), or mammalian-type NO synthase (NOS) or its inhibitors including l-*N* ^G^-nitro arginine methylester (l-NAME) (Huang *et al.*, 2002; Polverari *et al.*, 2003; Parani *et al.*, 2004; Grün *et al.*, 2006; Palmieri *et al.*, 2008; Besson-Bard *et al.*, 2009). Recent studies sshowed inconsistent findings concerning the effects of these NOS inhibitors, indicating that these chemicals have different or even opposite metabolic effects, and care must be taken in making inferences based on the use of these NO-modulating compounds (Arasimowicz-Jelonek *et al.*, 2011; Gupta *et al.*, 2011).

Several recent reports documented that the constitutive expression of rat nNOS in transgenic *Arabidopsis* plants resulted in the accumulation of endogenous NO and increased tolerance to abiotic and biotic stresses (Shi et al., 2012*b*, *c*). Similarly, Chun *et al.* (2013) introduced rat nNOS into tobacco plants and found that nNOS transgenic plants with overproduction of NO exhibited enhanced resistance to bacteria, fungi, and viruses. The use of nNOS transgenic *Arabidopsis* plant represents a new approach to study the effect of NO. In this system, NO is released *in planta* as a consequence of the constitutive expression of mammalian nNOS (Shi et al., 2012*b*, *c*; Chun *et al.*, 2013).

To gain insight into NO-mediated stress tolerance, nNOS transgenic *Arabidopsis* plants with increased *in vivo* NO content were used for physiological and transcriptomic analyses in the current study. Physiological assays characterized the effects of increased *in planta* NO production on antioxidant enzyme activities, reactive oxygen species (ROS), and osmolyte accumulation under drought stress conditions. Transcriptomic analysis identified several stress-related genes and revealed related pathways that were significantly changed in the nNOS transgenic plants. Functional analyses of downstream NO-regulated genes, including those for two ABA receptors (*PYL4* and *PYL5*), indicated that they played important roles during drought stress response. This study increases our understanding of the physiological and molecular roles of NO in the response of *Arabidopsis* to drought stress.

## Materials and methods

### Plant materials and growth conditions

This study used two transgenic *Arabidopsis* lines (nNOS-2 and nNOS-25) with the nNOS gene (Shi *et al.*, 2012*c*) and also used *AtPYL4*- and *AtPYL5*-overexpressing plants under the control of the *Cauliflower mosaic virus* (CaMV) 35S promoter and Col-0 (wild type, WT). For the overexpression of *AtPYL4* and *AtPYL5*, *AtPYL4* and *AtPYL5* cDNAs were cloned into the pCAMBIA99-1 vector. Then the constructs were transformed into *Agrobacterium tumefaciens* strain GV3101 and introduced into *Arabidopsis* WT (Col-0) plants using the floral dip method. Homozygous transgenic plants were selected using hygromycin resistance and were confirmed by PCR analyses.

After stratification in deionized water at 4 °C for 3 d in darkness, the *Arabidopsis* seeds were sown in soil-filled plastic containers in a growth room. The growth room was maintained at 22–25 °C with an irradiance of 120–150 μmol quanta m^–2^ s^–1^, 65% relative humidity, and a 16h light/8h dark cycle. Nutrient solution was added twice each week.

### Drought stress treatment

To apply the drought stress treatment (via soil water deficit), water was withheld from 2-week-old WT and transgenic plants in soil for 21 d before the plants were re-watered. The survival rate of the stressed plants was recorded 7 d after re-watering. Leaf samples were harvested at day 7, 14, and 21 (referring to the number of days since the initiation of the drought stress treatment) under control and drought conditions for physiological parameter analyses.

### Quantification of NO content and plant growth parameters

The NO content in leaf samples was quantified using the haemoglobin method by examining the conversion of oxyhaemoglobin to methaemoglobin as previously described (Shi *et al.*, 2012*c*, 2013*a*). Plant height and dry weight (DW) were measured ~80 d after the seeds were sown.

### Determination of electrolyte leakage (EL) and leaf water content (LWC)

EL was determined as described by Shi et al. (2012*a*, 2013*a*, *b*). For determination of EL and LWC, leaf samples were harvested at 0, 7, 14, and 21 d after drought stress and 7 d after re-watering. Fresh weight (FW) was measured immediately after harvest, and the DW was measured after 16h at 80 °C. LWC (%) was measured as (FW–DW)/FW×100.

### Determination of proline, sucrose, and total soluble sugar levels

For determination of proline content, a 0.5g aliquot of each leaf sample was ground and extracted in 3% (w/v) sulphosalicylic acid before 2ml of ninhydrin reagent and 2ml of glacial acetic acid were added. The mixed solutions were boiled at 100 °C for 40min and cooled to room temperature. The proline level in the sample was calculated based on absorbance at 520nm and was expressed as μg per g FW of sample (Shi *et al.*, 2012*a*). For the determination of sucrose and total soluble sugars, the anthrone method was used as previously described (Shi *et al.*, 2012*a*).

### Determination of H_2_O_2_ content and antioxidant enzyme activities

For determination of H_2_O_2_ content and antioxidant enzyme activities, plant extracts were isolated in 50mM sodium phosphate buffer (pH 7.8) using materials harvested from drought-stressed and control plants at 7, 14, and 21 d. H_2_O_2_ content and the activities of antioxidant enzymes [superoxide dismutase (SOD; EC 1.15.1.1), catalase (CAT; EC 1.11.1.6), glutathione reductase (GR; EC 1.6.4.2), glutathione peroxidase (GPX; EC 1.11.1.9), and peroxidase (POD; EC 1.11.1.7)] were quantified using previously published protocols (Shi et al., 2012*a*, 2013*a*, *b*). The H_2_O_2_ content was expressed as μM per FW. The relative activities of these antioxidant enzymes were expressed as the fold change relative to the WT (Col-0) under control conditions at 7 d.

### RNA isolation, array hybridization, and microarray analysis

For RNA isolation, 2-week-old WT and nNOS transgenic plants in pots were well watered (control condition) or subjected to drought conditions by withholding water for 7 d. Each combination of genotype and treatment was represented by two replicate leaf samples, and each sample contained leaves from at least 20 seedlings. Total RNA was extracted with TRIzol reagent (Invitrogen) and was quantified as previously described (Shi *et al.*, 2012*c*). RNA quality was determined using a formaldehyde agarose gel and a 2100 Bioanalyzer (Agilent Technologies, Santa Clara, CA, USA) according to the manufacturer’s protocol.

Array hybridization and microarray analysis were performed by CapitalBio Corporation in China. For array hybridization, 200ng of total RNA was used for first-strand and second-strand cDNA synthesis. An equal amount of RNA from two independent nNOS transgenic lines (line 2 and line 25) was pooled for cRNA labelling. The cRNA was labelled with a biotinylated ribonucleotide analogue and was fragmented with fragmentation buffer using the MessageAmp™ Premier RNA Amplification Kit (Ambion, #1792). After purification, 12.5 μg of labelled and fragmented cRNA probes were hybridized to the *Arabidopsis* arrays with the Hybridization, Wash and Stain Kit (Affymetrix, #900720) according to the manufacturer’s instructions.

The arrays were scanned using a GeneChip^®^ Scanner 3000 (Affymetrix, #3000). The scanned images were saved as DAT files and were transformed to JPG images. The signal intensities were extracted from the JPG images with Affymetrix^®^ GeneChip^®^ Command Console^®^ Software (AGCC software) and were saved as CEL files. The affylmGUI package (Wettenhall *et al.*, 2006) rooted in R (Gentleman *et al.*, 2004) was used to calculate the intensity ratios and fold changes. All of the differentially expressed genes with *P*-values <0.05 and log_2_ fold change >1 or < –1 were chosen for further analysis. The normalized microarray data were submitted to the Gene Expression Omnibus (GEO) database with accession number GSE48474. All of the genes whose expression significantly changed in at least one comparison of genotype or drought treatment (*P*-value ≤0.05 and log_2_ fold change ≥1 or log_2_ fold change ≤ –1) are listed in Supplementary Table S1 available at *JXB* online.

### Quantitative real-time PCR

Total RNA was isolated from 100mg of leaves using 1ml of TRIzol reagent (Invitrogen). The total RNA was treated with RQ1 RNase-free DNase (Promega). RNA samples from two independent nNOS transgenic lines (line 2 and line 25) were pooled for cDNA synthesis. First-strand cDNA was synthesized with the RevertAid™ First Strand cDNA Synthesis Kit (Fermentas) using 2 μg of total RNA according to the manufacturer’s instructions. Reverse transcription products (cDNA) were diluted five times in water, and 2 μl of diluted cDNA was used for quantitative real-time PCR assay with iQ™ SYBR^®^ Green Super mix (Bio-Rad). Quantitative real-time PCR was performed using the CFX96™ Real Time System (Bio-Rad) with SYBR green fluorescence as previously described (Shi *et al.*, 2012*c*, 2013*a*, *b*). The experiment was performed with at least three independent replicates, and the comparative ΔΔCT method was used for comparative gene expression analysis. In total, 30 genes with a ≥2-fold change in expression were randomly selected for real-time PCR assay. The housekeeping gene *UBQ10* was used as an endogenous control. The primers used are listed in Table S2 at *JXB* online.

### Biological enrichment and metabolic pathway analyses

All differentially expressed genes with *P*-values ≤0.05 and log_2_ fold change ≥1 or ≤ –1 were loaded and annotated in the Classification SuperViewer Tool (http://bar.utoronto.ca/ntools/cgi-bin/ntools_classification_superviewer.cgi) (Provart and Zhu, 2003). Functional categories of every gene and pathway were assigned using MapMan (http://mapman.mpimp-golm.mpg.de/general/ora/ora.html) as the classification source (Thimm *et al.*, 2004). Additionally, the normalized frequency (NF) of each functional category was calculated as follows: NF=sample frequency of each category in this experiment⁄background frequency of each category in the ATH1 array. For GO term enrichment analysis, differentially expressed genes were input into the agriGO website (http://bioinfo.cau.edu.cn/agriGO/index.php), and the Singular Enrichment Analysis (SEA) tool was used for enrichment analyses (Du *et al.*, 2010).

### Hierarchical cluster analysis

The data sets of specific genes were imported for hierarchical cluster analysis, which was performed using an uncentred matrix and complete linkage method with the CLUSTER program (http://bonsai.ims.u-tokyo.ac.jp/~mdehoon/software/cluster/) (de Hoon *et al.*, 2004). Resulting tree figures were displayed using the software package Java Treeview (http://jtreeview.sourceforge.net/) as described by Chan *et al.* (2012) and Chan (2012).

### Statistical analysis

All of the experiments in this study were repeated three times, and the values presented are means ±SEs. For each independent experiment, each leaf sample extract was derived from the leaves of at least 15 plants. Asterisks above the columns in figures indicate significant differences relative to the WT at *P*<0.05 (Duncan’s multiple range test).

## Results

### Growth of nNOS transgenic and WT plants under control and drought conditions

Under well-watered (control) conditions, growth of nNOS transgenic plants (line 2 and line 25) was generally equivalent to that of the WT (Supplementary Fig. S1a at *JXB* online). Under water deficit conditions, growth of both nNOS transgenic lines and WT plants was inhibited, as previously reported (Shi *et al.*, 2012*c*), but nNOS transgenic plants (line 2 and line 25) had greener leaves than WT plants (Supplementary Fig. S1a). Consistent with previous results (Shi *et al.*, 2012*c*), nNOS transgenic plants accumulated higher concentrations of endogenous NO under both control and drought stress conditions (Supplementary Fig. S1b).

**Fig. 1.**
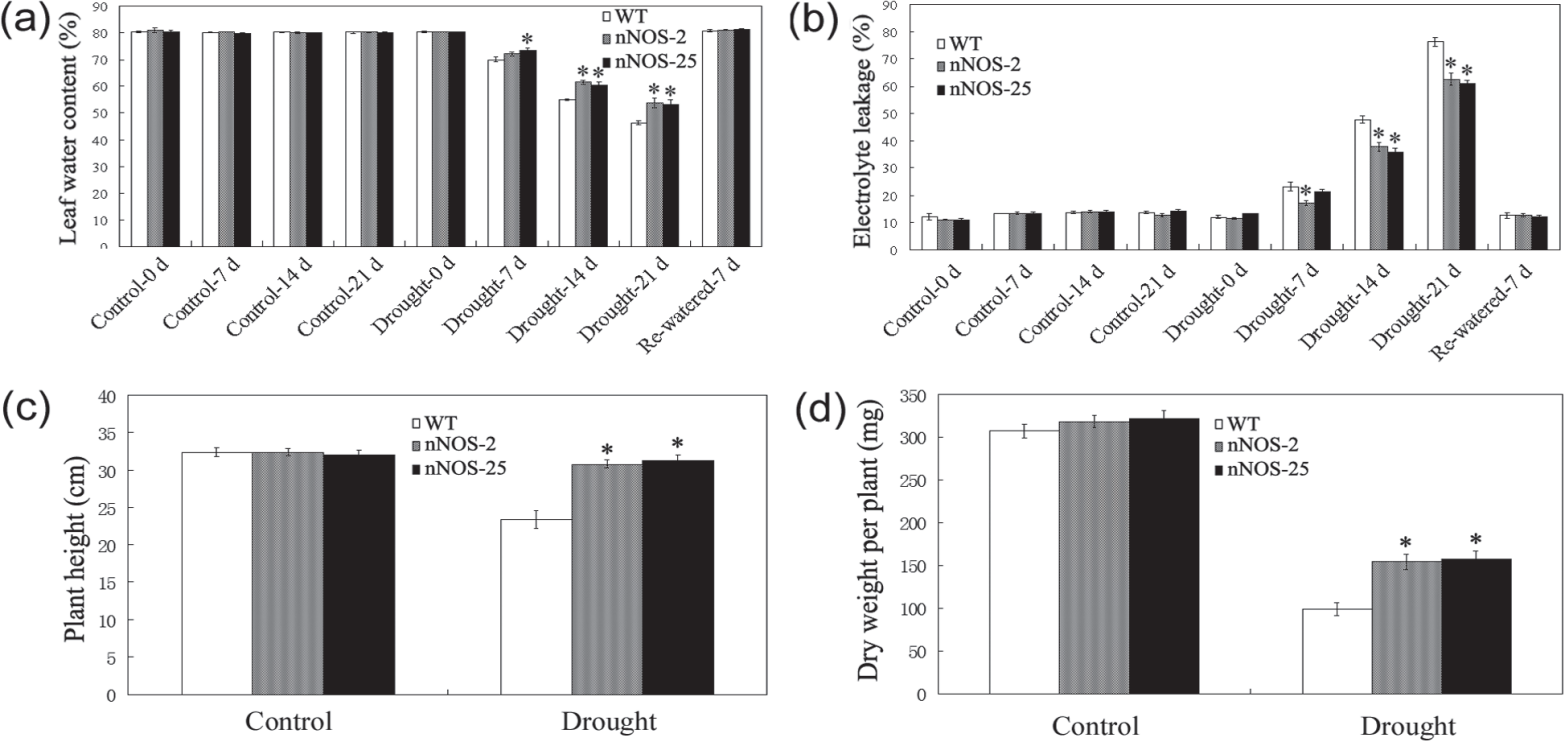
Performance of WT and nNOS transgenic *Arabidopsis* plants under drought stress conditions (soil water deficit). (a and b) LWC (a) and EL (b) of WT and nNOS transgenic plants during control and drought stress conditions. (c and d) Plant height (c) and dry weight (DW) (d) of WT and nNOS transgenic plants under control and drought stress conditions at harvest. Values are means ±SEs (*n*=4 for a, b, *n*=20 for c, d). Asterisks indicate significant differences between WT and nNOS transgenic plants (*P*<0.05).

Under control conditions, both nNOS transgenic lines and WT plants maintained LWC at ~80% (Fig. 1a). After drought stress treatment, the LWC gradually declined in all plants, but the decline was greater in the WT than in the nNOS transgenic plants (Fig. 1a). Consistent with the decline in LWC, all plants showed a gradual increase in EL after drought stress treatment, but the increase was less in the nNOS transgenic lines than in the WT (Fig. 1b).

After re-watering for 7 d, almost all WT plants died, while >55% of the nNOS plants survived (Supplementary Fig. S1a at *JXB* online). Among the surviving WT and nNOS transgenic plants, LWC and EL were recovered, and there were no significant differences between WT and nNOS transgenic plants in LWC and EL (Fig. 1a, b). At harvest time (i.e. ~45 d after re-watering), plant height and biomass (DW) of plants subjected to water deficit conditions were greater in nNOS transgenic lines than in the WT (Fig. 1c, d).

### Osmolyte accumulation and ROS metabolism in nNOS transgenic and WT plants under drought stress

**Fig. 2.**
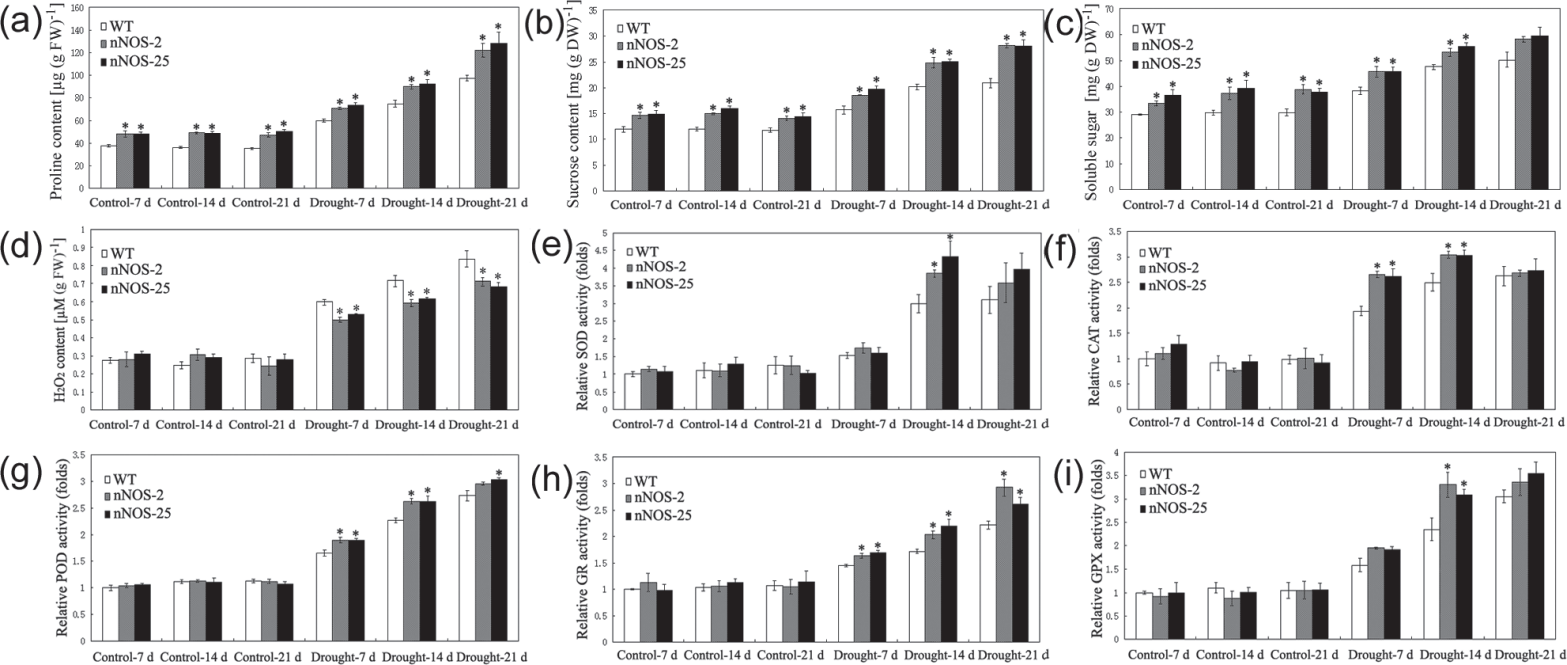
Osmolyte levels and ROS metabolism in WT and nNOS transgenic plants during drought stress. (a–i) Proline content (a), sucrose content (b), soluble sugar content (c), H_2_O_2_ content (d), and activities of SOD (e), CAT (f), POD (g), GR (h), and GPX (i) in WT and nNOS transgenic plants under control and drought stress conditions. The relative activities were quantified as the fold change relative to the activity in the WT under control conditions at 7 d. Values are the means ±SEs (*n*=4). Asterisks indicate significant differences between WT and nNOS transgenic plants (*P*<0.05).

Under control conditions, both nNOS transgenic lines (nNOS-2 and nNOS-25) accumulated significantly higher levels of proline, sucrose, and total soluble sugars than the WT (Fig. 2a–c). Drought stress increased the levels of proline, sucrose, and total soluble sugars in both nNOS transgenic lines and WT plants, but the increase was greater in the nNOS transgenic lines than in the WT (Fig. 2a–c).

As the major indicator of ROS level and oxidative damage, H_2_O_2_ functions as the key stress-related signal, and H_2_O_2_ content was assayed in this study. Under control conditions, H_2_O_2_ levels did not significantly differ in the nNOS transgenic lines and the WT plants (Fig. 2d). After 7, 14, and 21 d of drought stress treatment, however, H_2_O_2_ content was significantly lower in the nNOS transgenic lines than in the WT (Fig. 2d). Under control conditions, the activities of five antioxidant enzymes (SOD, CAT, POD, GR, and GPX) did not significantly differ in nNOS transgenic lines and the WT (Fig. 2e–i). After drought stress treatment, however, the activities of the five enzymes were significantly higher at several time points in nNOS transgenic lines than in the WT (Fig. 2e–i).

### Differentially expressed genes in WT and nNOS transgenic plants under control and drought conditions

**Fig. 3.**
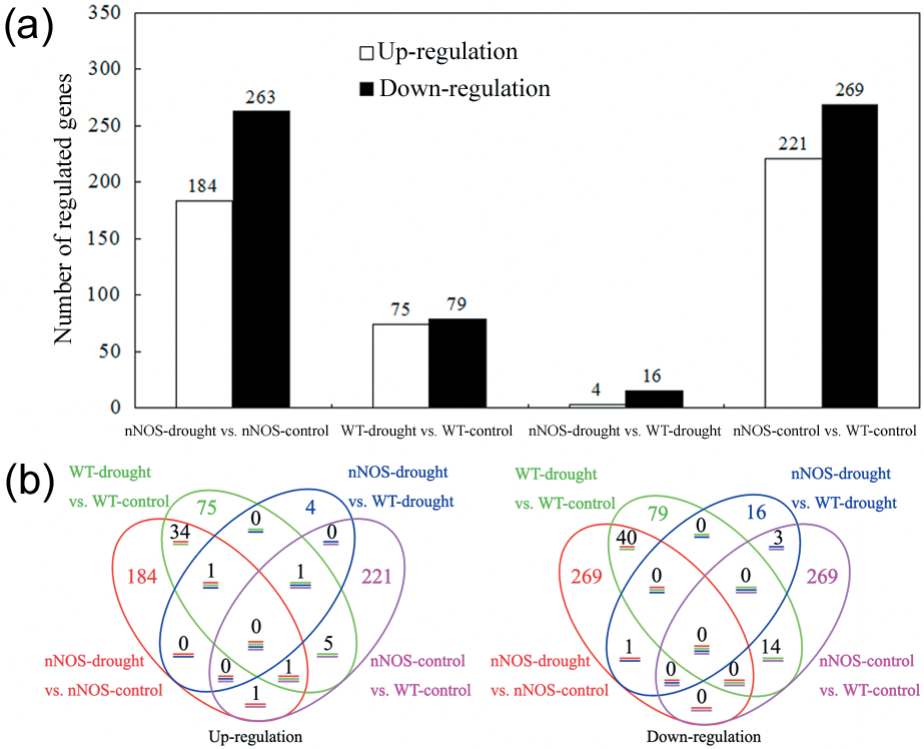
Number of genes differentially expressed in WT versus nNOS transgenic plants under control and drought conditions. (a) Total number of affected genes in WT and nNOS transgenic plants under control and drought conditions. (b) Venn diagram showing the number of overlapping genes that are differentially expressed between WT and nNOS transgenic plants under control and drought conditions. (This figure is available in colour at *JXB* online.)

Microarray analysis was performed to gain insight into NO-mediated drought stress responses. In total, the expression levels of 154 genes (75 were up-regulated genes and 79 were down-regulated genes) in WT plants and 447 genes (184 were up-regulated genes and 263 were down-regulated genes) in nNOS transgenic plants were changed by at least 2-fold in response to drought stress treatment (Fig. 3a; Supplementary Table S1 at *JXB* online). Under control conditions, 490 genes differed in expression level in nNOS transgenic plants compared with the WT, comprising 221 up-regulated and 269 down-regulated genes (Fig. 3a). Under drought conditions, 20 genes differed in expression level in nNOS transgenic plants compared with the WT, comprising four up-regulated and 16 down-regulated genes (Fig. 3a). As shown in Fig. 3b, only a small number of differentially expressed genes were common to all four comparisons, including two comparisons for genotype effect (nNOS control versus WT control, nNOS drought versus WT drought) and two comparisons for stress effect (WT drought versus WT control, nNOS drought versus nNOS control). However, 30% of the genes changed by drought stress in the WT were also changed by drought stress in the nNOS transgenic plants (Fig. 3b). Thirty genes regulated by drought in the WT (WT drought versus WT control) and by the nNOS transgene effect in the absence of drought stress (nNOS control versus WT control) were also identified (Table 1). These genes might play direct roles in nNOS transgene-induced drought stress tolerance. Interestingly, only two genes were regulated in common in nNOS control versus WT control and nNOS drought versus nNOS control (Fig. 3b), while 155 genes were oppositely regulated in these two comparisons (Supplementary Table S1).

**Table 1.** Genes highly regulated by drought stress and by the nNOS transgenic effect in *Arabidopsis*

AGI	nNOS drought versus nNOS control		WT drought versus WT control		nNOS drought versus WT drought		nNOS control versus WT control		Description	MapMAN Bin name
	log_2_	*P*-value	log_2_	*P*-value	log_2_	*P*-value	log_2_	*P*-value		
At4g25100	**1.23**	**0.0000**	**2.13**	**0.0000**	0.21	0.2317	**1.11**	**0.0001**	Fe-superoxide dismutase	Redox. dismutases and catalases
At5g53870	–0.02	0.9214	**1.42**	**0.0001**	0.12	0.5431	**1.57**	**0.0000**	Early nodulin-like protein 1	Misc. plastocyanin-like
At2g35980	0.26	0.1609	**1.30**	**0.0002**	0.06	0.7789	**1.10**	**0.0002**	NDR1/HIN1-LIKE 10	Stress. biotic
At1g24140	–0.03	0.8622	**1.20**	**0.0002**	0.22	0.2381	**1.45**	**0.0000**	Matrixin family protein	Protein. degradation.metalloprotease
At4g27654	–0.18	0.5689	**1.14**	**0.0083**	1.10	0.0138	**2.41**	**0.0001**	Unknown protein	Not assigned. unknown
AtCg00690	0.51	0.1258	**1.14**	**0.0125**	0.78	0.0524	**1.40**	**0.0012**	5kDa protein subunit PSII-T	PS. Light reaction. photosystem II
At1g23040	0.05	0.7613	**1.02**	**0.0003**	0.14	0.4071	**1.10**	**0.0001**	Hydroxyproline-rich glycoprotein	No ontology.hydroxyproline rich proteins
At1g75940	–0.01	0.9742	*–1.07*	*0.0087*	–0.28	0.3655	*–1.33*	*0.0007*	beta-Glucosidase 20	Misc. gluco-, galacto- and mannosidases
At1g57750	0.90	0.0002	*–1.11*	*0.0002*	–0.60	0.0116	*–2.61*	*0.0000*	CYP96A15	Not assigned. unknown
At3g49620	–0.06	0.7921	*–1.15*	*0.0011*	–0.63	0.0245	*–1.72*	*0.0001*	DIN11	Development. unspecified
At1g11580	–0.17	0.3290	*–1.24*	*0.0002*	0.00	0.9973	*–1.07*	*0.0001*	Pectin methylesterase	Cell wall. pectin esterases.PME
At5g07550	0.40	0.0342	*–1.33*	*0.0001*	–0.58	0.0177	*–2.31*	*0.0000*	GRP19	No ontology. glycine rich proteins
At2g39330	0.15	0.6476	*–1.37*	*0.0035*	–0.30	0.3505	*–1.82*	*0.0002*	JAL23	Misc. myrosinases-lectin-jacalin
At2g45130	–0.41	0.0273	*–1.39*	*0.0001*	–0.03	0.8772	*–1.01*	*0.0002*	SPX3	Stress. abiotic
At3g25050	–0.03	0.9571	*–1.48*	*0.0095*	0.00	0.9992	*–1.45*	*0.0028*	XTH3	Cell wall. modification
At1g17710	–0.29	0.2000	*–1.74*	*0.0001*	0.07	0.7900	*–1.38*	*0.0002*	Phosphocholine phosphatase	Misc. acid and other phosphatases
At1g73010	–0.78	0.0012	*–1.85*	*0.0000*	–0.46	0.0372	*–1.53*	*0.0000*	Pyrophosphate-specific phosphatase	Misc. acid and other phosphatases
At1g54020	0.09	0.7179	*–1.95*	*0.0001*	–0.22	0.3571	*–2.26*	*0.0000*	Myrosinase-associated protein	Secondary metabolism. degradation
At1g66850	0.77	0.0010	*–2.03*	*0.0000*	–0.73	0.0094	*–3.54*	*0.0000*	Lipid-transfer protein	Misc. protease inhibitor
At5g20790	–0.93	0.0010	*–2.39*	*0.0000*	0.09	0.6912	*–1.36*	*0.0001*	Unknown protein	Not assigned. unknown
At1g72260	–0.08	0.7225	*–2.73*	*0.0000*	0.38	0.1165	*–2.26*	*0.0000*	THI2.1	Stress. biotic.receptors
At5g45890	**1.75**	**0.0000**	**1.27**	**0.0009**	**–0.92**	0.0103	*–1.41*	*0.0002*	SAG12	Protein. degradation.cysteine protease
At2g17880	*–1.93*	*0.0000*	*–1.03*	*0.0006*	*0.12*	0.5189	**1.01**	**0.0002**	DNAJ heat shock protein,	Stress. abiotic.heat
At3g28310	*–2.22*	*0.0000*	*–1.15*	*0.0012*	*0.03*	0.9057	**1.10**	**0.0004**	Unknown protein	Not assigned. no ontology
At5g56100	*–2.12*	*0.0000*	*–1.31*	*0.0001*	*0.52*	0.0225	**1.34**	**0.0001**	Glycine-rich protein	Lipid metabolism. TAG synthesis
At4g12500	*–3.42*	*0.0000*	*–1.61*	*0.0000*	*–0.20*	0.2350	**1.62**	**0.0000**	Lipid-transfer protein	Misc. protease inhibitor
At1g15010	*–2.32*	*0.0000*	*–1.67*	*0.0000*	*0.69*	0.0103	**1.34**	**0.0001**	Unknown protein	Not assigned. unknown
At2g38310	–0.95	0.0002	–0.33	0.0699	0.50	0.0187	**1.12**	**0.0000**	PYL4/RCAR10	Stress. abiotic
At5g05440	*–2.40*	*0.0000*	*–1.75*	*0.0000*	0.55	0.0166	**1.21**	**0.0001**	PYL5/RCAR8	Stress. abiotic
At1g19610	*–4.01*	*0.0000*	*–1.78*	*0.0000*	–0.14	0.4867	**2.08**	**0.0000**	Pathogenesis-related protein	Stress. biotic

Genes highly regulated by drought stress and by the nNOS transgenic effect (i.e. with a *P*-value ≤0.05 and log_2_ fold change ≥1 or log_2_ fold change ≤ –1) were classified using MapMan.

Values in bold indicate significant up-regulation, and those in italics indicate significant down-regulation.

To confirm the reliability of the microarray data, the expression of 30 genes that were differentially expressed between WT and nNOS transgenic plants was assessed via quantitative real-time PCR. Although several genes (only four genes) showed a <2-fold change in real-time PCR, the results of the real-time PCR assay exhibited the same trend and were correlated with the microarray data (*R* ^2^=0.8951) (Supplementary Fig. S2a–c at *JXB* online), confirming the reproducibility of microarray analysis.

### Cluster and pathway enrichment analyses of differentially expressed genes changed by nNOS transgene or by drought

Pathway analysis revealed that the nNOS transgene and drought affected the expression of many genes involved in photosynthesis, hormone metabolism, redox, stress, mitochondrial electron transport, oxidative pentose phosphate (OPP), and lipid metabolism (Table 2, group I). Further analyses showed that many redox- and phytohormone metabolism-related genes were extensively regulated by the nNOS transgene effect (Supplementary Fig. S3a, b at *JXB* online). Interestingly, genes involved in ABA, indole-3-acetic acid (IAA), benzylaminopurine (BA), and ethylene metabolism were constitutively up-regulated in nNOS transgenic plants (Supplementary Fig. S3a, b). Moreover, ectopic expression of the nNOS gene in *Arabidopsis* also resulted in activation of the light reaction and sugar biosynthesis pathways (Supplementary Fig. S4). Several other pathways including development, major carbohydrate (CHO) metabolism, protein, signalling, and cell wall were over-represented in nNOS trangenic versus WT plants under control conditions (nNOS control versus WT control) but under-represented in nNOS transgenic versus WT plants under drought conditions (nNOS drought versus WT drought) (Table 2, group II).

**Table 2.** Pathway enrichment analysis of genes whose expression was significantly affected by drought stress and by the nNOS transgenic effect in *Arabidopsis*

Groups	Pathways	nNOS drought versus nNOS control		WT drought versus WT control		nNOS drought versus WT drought		nNOS control versus WT control	
		NF	*P*-value	NF	*P*-value	NF	*P*-value	NF	*P*-value
I	PS	**5.49**	**0.0000**	**2.11**	0.1740	**8.15**	0.1090	**6.68**	**0.0000**
	Metal handling	**4.54**	**0.0041**	**15.77**	**0.0000**	–	–	–	–
	Hormone metabolism	**4.18**	**0.0000**	**2.41**	**0.0260**	–	–	**1.52**	**0.0440**
	Redox	**2.15**	**0.0390**	**11.4**	**0.0000**	–	–	**1.96**	**0.0520**
	Stress	**2.09**	**0.0000**	**3.37**	**0.0000**	**2.73**	0.1300	**1.45**	**0.0140**
	Miscellaneous	**1.66**	**0.0011**	**3.7**	**0.0000**	**4.22**	0.0110	**1.34**	**0.0200**
	Mitochondrial electron transport	**1.50**	0.1820	**1.44**	0.3480	–	–	**2.28**	**0.0470**
	Oxidative Pentose phosphate	**2.43**	0.2750	**7.03**	0.1240	–	–	**2.22**	0.2900
	Lipid metabolism	**1.05**	0.1610	**1.01**	0.2730	–	–	**1.12**	0.1440
II	Secondary metabolism	**1.53**	0.0650	**0.98**	0.2730	–	–	**2.48**	**0.0006**
	RNA	**1.48**	**0.0005**	**0.64**	**0.0450**	**1.65**	0.1690	**1.01**	0.0620
	Transport	**1.40**	**0.0330**	**1.7**	**0.0520**	–	–	**0.53**	**0.0200**
	Development	**0.95**	0.1260	**1.38**	0.1400	**4.26**	0.0680	**1.39**	**0.0440**
	Cofactor and vitamin metabolism	**0.93**	0.3700	–	–	–	–	**3.4**	**0.0240**
	Major CHO metabolism	**0.74**	0.3530	–	–	–	–	**2.72**	**0.0440**
	Protein	**0.7**	**0.0016**	**0.54**	**0.0049**	**0.69**	0.2400	**1.08**	**0.0380**
	Signalling	**0.66**	**0.0350**	**0.64**	0.1220	**1.23**	0.3700	**1.11**	**0.0770**
	Amino acid metabolism	**0.58**	0.1890	**0.83**	0.3640	–	–	**1.58**	0.0930
	Cell wall	**0.41**	**0.0440**	**1.19**	0.2170	–	–	**1.88**	**0.0081**
	Cell	**0.36**	**0.0095**	**0.52**	0.1600	**6.07**	0.0110	**0.66**	0.0630
III	Not assigned	**0.93**	**0.0240**	**0.53**	**0.0000**	**0.56**	0.0730	**0.89**	**0.0096**
	DNA	**0.07**	**0.0000**	**0.13**	**0.0000**	–	–	**0.11**	**0.0000**

Differentially expressed genes (i.e. with *P* value ≤0.05 and log_2_ fold-change ≥1 or log_2_ fold-change ≤ –1) were annotated using the Classification SuperViewer Tool and MapMan. shading scales of NF are as follows:

**≥3 2–3 1–2 0.5–1 ≤0.5**

Not surprisingly, GO term enrichment analysis showed that many metabolic-, ion homeostasis-, and transport-related pathways were extensively changed after drought stress treatment, resulting in enrichment of stress-responsive GO terms (Supplementary Fig. S5a at *JXB* online). Comparatively, transformation of *Arabidopsis* with the nNOS gene changed nitrogen metabolism as expected, as well as photosynthesis, energy-producing, and phytohormone metabolism pathways. These changes might contribute to the enrichment of stress-related GO terms (Supplementary Fig. S5b).

**Fig. 4.**
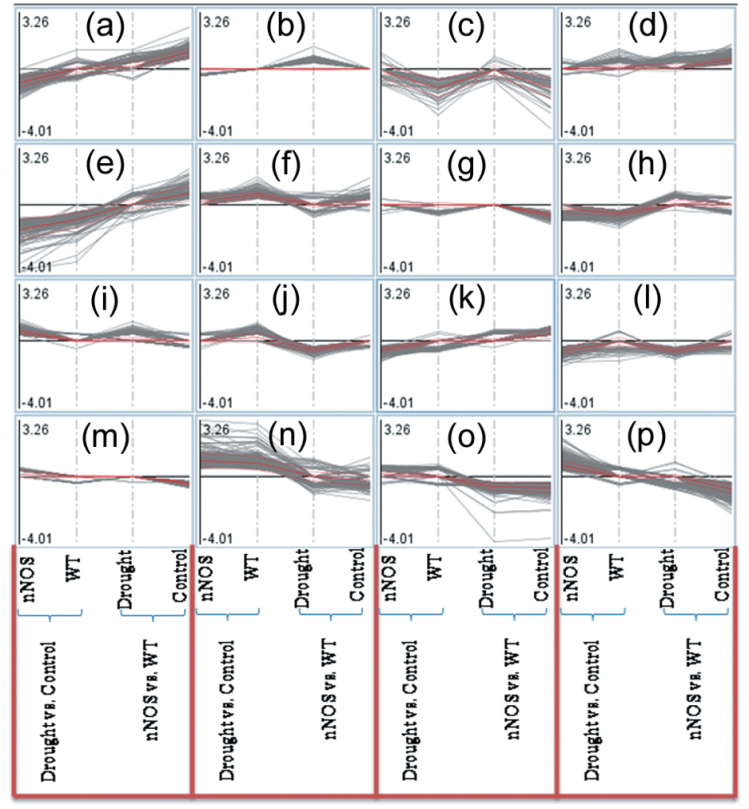
Cluster analysis of microarray data using MapMan software. All microarray data were divided into 16 clusters that were labelled from (a) to (p). Detailed information for each cluster is provided in Supplementary Table S3 at JXB online. (This figure is available in colour at *JXB* online.)

Genome-wide cluster analysis indicated that genes in cluster d were mainly up-regulated, while those in cluster l were mainly down-regulated by both the nNOS transgene and drought stress treatment. In addition, genes in clusters e and h were mainly up-regulated by the nNOS transgene but down-regulated by the drought stress treatment, and genes in clusters n and o were mainly up-regulated by drought stress treatment but down-regulated by the nNOS transgene (Fig. 4; Table S3 at *JXB* online).

### Comparison of genes whose expression is altered by the nNOS transgene and NO donor treatment

Genes whose expression was affected by both the nNOS transgene and by NO donor (SNP) treatment were also identified (Ahlfors *et al.*, 2009). Among the 28 genes whose expression was affected by both factors, 21 were up-regulated and one was down-regulated by both treatments, while six others differed in expression pattern due to the nNOS transgene and SNP treatment (Table 3).

**Table 3.** Genes that were differentially expressed in response to both the nNOS transgenic effect and the NO donor (SNP) effect in *Arabidopsis*

ID	Description	nNOS^*a*^ versus WT	SNP^*b*^ versus control	MapMAN	
				Bin code	Bin name
At3g44720	ADT4 (arogenate dehydratase 4)	**1.18**	**1.21**	13.1.6	Amino acid metabolism. synthesis
At4g35630	PSAT; *O*-phospho-l-serine:2-oxoglutarate aminotransferase	*–1.11*	0.98	13.1.5	Amino acid metabolism. synthesis
At4g09030	AGP10 (ARABINOGALACTAN PROTEIN 10)	**1.11**	**1.12**	10.5.1	Cell wall. cell wall proteins. AGPs
At1g19300	Polygalacturonate 4-alpha-galacturonosyltransferase	**1.34**	**1.04**	10.3.2	Cell wall. hemicellulose synthesis. glucuronoxylan
At2g38360	PRA1.B4 (PRENYLATED RAB ACCEPTOR 1.B4)	**1.14**	**0.96**	31.4	Cell. vesicle transport
At5g65870	ATPSK5 (PHYTOSULPHOKINE 5 PRECURSOR)	**1.20**	**1.64**	33.99	Development. unspecified
At3g15210	RAP2.5/ERF4	**1.39**	**1.74**	17.5.2	Hormone metabolism. ethylene.signal transduction
At1g23440	Pyrrolidone-carboxylate peptidase family protein	–0.54	**1.03**	29.5	Protein. degradation
At1g24140	Matrixin family protein	**1.45**	**1.88**	29.5.7	Protein. degradation. metalloprotease
At5g27420	Zinc finger (C3HC4-type RING finger) family protein	**1.49**	**2.98**	29.5.11	Protein. degradation. ubiquitin. E3. RING
At4g35480	RING-H2 finger protein RHA3b	**1.24**	**1.59**	29.5.11	Protein. degradation. ubiquitin. E3. RING
At5g47610	Zinc finger (C3HC4-type RING finger) family protein	**1.21**	*–1.01*	29.5.11	Protein. degradation. ubiquitin. E3. RING
At5g66070	Zinc finger (C3HC4-type RING finger) family protein	**1.02**	**1.83**	29.5.11	Protein. degradation. ubiquitin. E3. RING
At5g47070	Protein kinase, putative	*–1.08*	**1.39**	29.4.1	Protein. postranslational modification. kinase
At1g28480	GRX480; electron carrier/protein disulfide oxidoreductase	**1.15**	**1.46**	21.4	Redox. glutaredoxins
At5g22250	CCR4-NOT transcription complex protein, putative	**1.31**	**1.53**	27.1.19	RNA. processing. ribonucleases
At1g27730	ZAT10/STZ (salt tolerance zinc finger)	**1.15**	**2.66**	27.3.11	RNA. regulation of transcription. zinc finger family
At5g54490	PBP1 (PINOID-BINDING PROTEIN 1)	**1.11**	**2.39**	30.3	Signalling. calcium
At3g01830	Calmodulin-related protein, putative	**1.00**	**2.95**	30.3	Signalling. calcium
At4g36040	DNAJ heat shock N-terminal domain-containing protein (J11)	**1.00**	**0.70**	20.2.1	Stress. abiotic.heat
At1g72940	Disease resistance protein (TIR-NBS class)	*–1.05*	0.77	20.1.7	Stress. biotic. PR-proteins
At5g52760	Heavy-metal-associated domain-containing protein	**1.01**	**1.33**	35.1	Not assigned. no ontology
At3g04640	Glycine-rich protein	**1.22**	**1.55**	35.1.40	Not assigned. no ontology. Glycine-rich proteins
At1g56060	Unknown protein	**1.07**	**2.48**	35.2	Not assigned. unknown
At2g25735	Unknown protein	**1.05**	**1.84**	35.2	Not assigned. unknown
At2g28400	Unknown protein	**1.02**	**2.38**	35.2	Not assigned. unknown
At5g53420	Unknown protein	*–1.02*	0.77	35.2	Not assigned. unknown
At1g13650	18S pre-ribosomal assembly protein gar2-related	*–1.18*	*–1.04*	35.2	Not assigned. unknown

^*a*^ The data for nNOScontrol versus WT control are from this study.

^*b*^ The data for log_2_ fold change of SNP 3h versus 0h are from Ahlfors *et al.* (2009).

Values in bold indicate significant up-regulation, and those in italics indicates significant down-regulation.

**Fig. 5.**
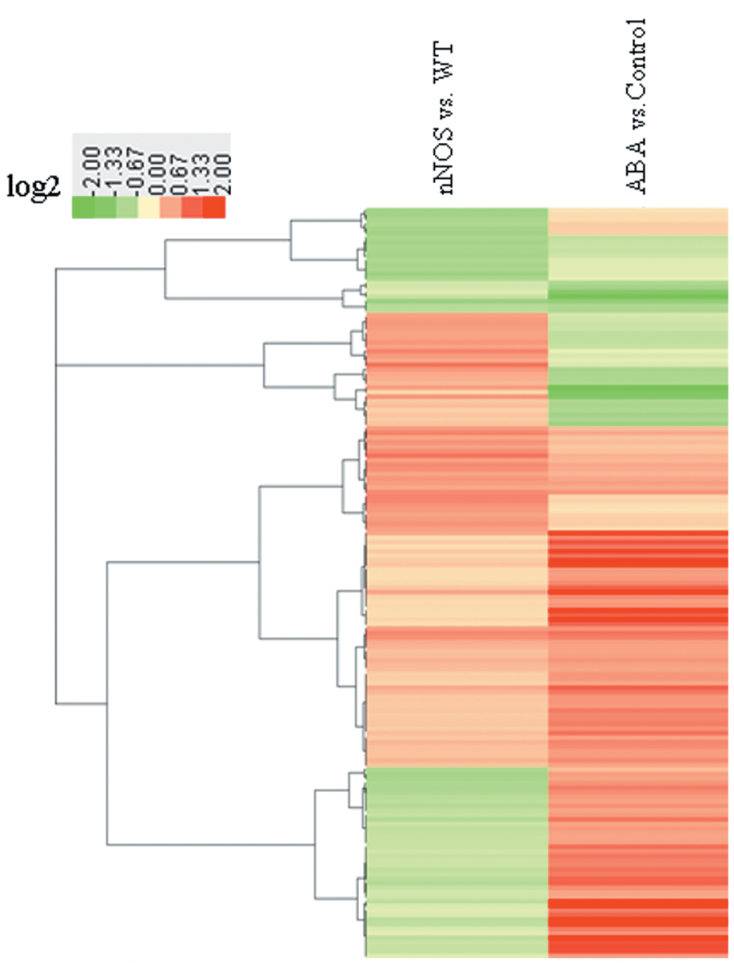
Hierarchical cluster analysis of genes regulated both by the nNOS transgene and by ABA treatment in *Arabidopsis*. The expression data for genes commonly regulated by nNOS and ABA were imported for cluster analysis, and the resulting tree figure was displayed using the software package and Java Treeview. The detailed information is provided in Supplementary Table S4 at *JXB* online. (This figure is available in colour at *JXB* online.)

Additionally, based on published data (Goda *et al.*, 2008), 165 genes that were regulated by both the nNOS transgenic effect and ABA treatment were found (Supplementary Table S4 at *JXB* online). Cluster analysis revealed that most of these genes were up-regulated or down-regulated by both the nNOS transgene and ABA effect (Fig. 5). Since ABA plays a critical role in plant drought stress response, further experiments were carried out to characterize putative connections between ABA and NO signalling pathways.

### Overexpression of *AtPYL4* and *AtPYL5* enhances drought tolerance

**Fig. 6.**
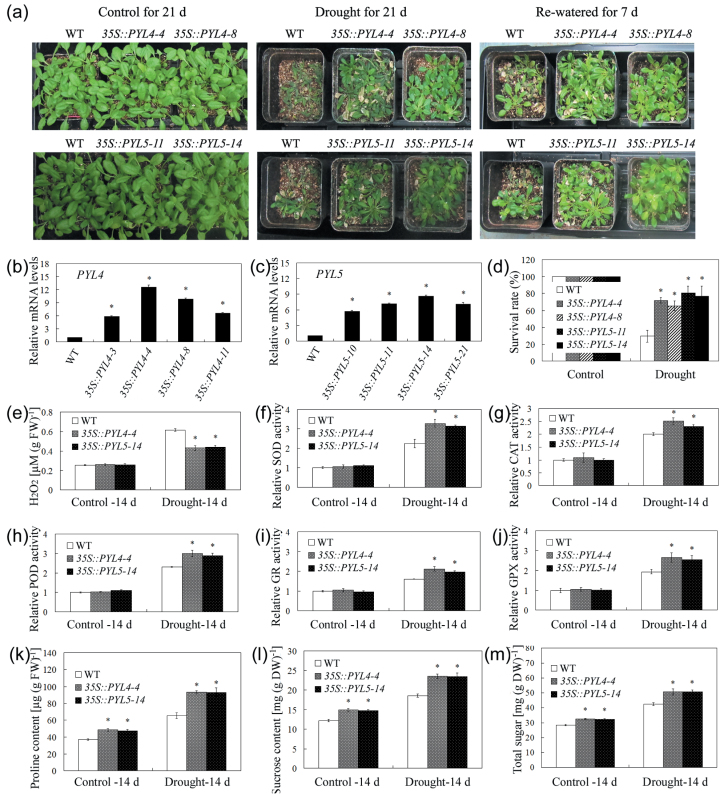
Enhanced drought resistance of plants overexpressing *AtPYL4* and *AtPYL5*. (a) Two-week-old plants were subjected to drought stress conditions (or to well-watered, control conditions) for 21 d before they were re-watered; the plants were photographed 7 d after watering was resumed. (b and c) Gene expression of *AtPYL4*- (b) and *AtPYL5*- (c) overexpressing plants. The relative mRNA level of WT plants was set at 1.0. (d) Survival of *AtPYL4*- and *AtPYL5*-overexpressing plants under control and drought stress conditions. (e–m) H_2_O_2_ content (e), SOD activity (f), CAT activity (g), POD activity (h), GR activity (i) GPX activity (j), proline content (k), sucrose content (l), and soluble sugar content (m) of WT, *AtPYL4*, and *AtPYL5* transgenic plants during control and drought stress conditions. The relative activities were quantified as fold change relative to the WT under control conditions for 14 d. Values are means ±SEs (*n*=3). Asterisks indicate significant differences between WT and nNOS transgenic plants (*P*<0.05). (This figure is available in colour at *JXB* online.)

To characterize further the *in vivo* roles of some differentially expressed genes in the nNOS transgenic lines, two ABA receptor genes (*AtPYL4/RCAR10* and *AtPYL5/RCAR8*), which act upstream of the ABA pathway and directly modulate many downstream genes (Table 1), were constitutively overexpressed in *Arabidopsis* (Fig. 6a–c). *AtPYL4*- and *AtPYL5*-overexpressing transgenic plants exhibited enhanced drought resistance (Fig. 6a–d). Under drought stress conditions, H_2_O_2_ levels were lower and activities of antioxidant enzymes (SOD, CAT, POD, GR, and GPX) were higher in *AtPYL4*- and *AtPYL5*-overexpressing plants than in the WT (Fig. 6e–j). Additionally, *AtPYL4*- and *AtPYL5*-overexpressing plants accumulated higher levels of proline, sucrose, and soluble sugars than the WT under both control and drought stress conditions (Fig. 6l, m). These results indicated that *AtPYL4* and *AtPYL5* enhanced drought tolerance, in part by modulating ROS metabolism and osmolyte levels.

## Discussion

In this study, comparative physiological and transcriptomic analyses (Huang *et al.*, 2011; Chan *et al.*, 2012) were used to assess the drought stress response of transgenic *Arabidopsis* plants that ectopically expressed the rat nNOS gene, which resulted in the specific release of NO *in planta*. Although mutants with increased NO content are available, some (such as *cue1/nox1* and *gsnor*) negatively affect plant development and yield (He *et al.*, 2004; Chen *et al.*, 2009), while others have a non-specific role in NO synthesis; *argah1/2* mutants, for example, modulate not only NO accumulation but also polyamine accumulation and arginine metabolism (Shi and Chan, 2013; Shi *et al.*, 2013*a*). In addition to investigating NO-mediated physiological responses, this study also partially revealed the transcriptomic modulations caused by constitutive NO release. Importantly, this study avoided the side effects caused by the use of NO-modulator compounds (Arasimowicz-Jelonek *et al.*, 2011; Gupta *et al.*, 2011), and characterized functions of some genes (such as *PYL4* and *PYL5*) induced in the nNOS transgenic plants, but not by SNP application (Ahlfors *et al.*, 2009).

Consistent with the results reported previously (Shi et al., 2012*b*, *c*), nNOS transgenic plants exhibited significantly improved drought stress resistance (Fig. 1; Supplementary Fig. S1 at *JXB* online), which might be attributed to the increased *in vivo* NO content. Additionally, the effect of re-watering (Xu *et al.*, 2010; Ziogas *et al.*, 2013) was also determined in this study. After re-watering for 7 d, almost all WT plants died, while >55% of the nNOS plants survived (Supplementary Fig. S1a). Although no significant differences of LWC and EL were observed between the surviving WT and nNOS transgenic plants after re-watering for 7 d, the drought stress effect was fully recovered, as nNOS transgenic lines exhibited higher plant height and more biomass (DW) at harvest time (~45 d after re-watering) (Fig. 1). These results indicated that re-watering recovered the water status and cell damage caused by drought stress, and nNOS transgenic plants exhibited improved drought resistance with a higher survival rate. Under control and drought stress conditions, nNOS transgenic plants accumulated higher levels of osmolytes (proline, sucrose, and total soluble sugars) relative to WT plants (Fig. 2a–c), which would protect *Arabidopsis* plants by increasing cell membrane stability and balancing osmotic pressure in response to drought stress. Additionally, nNOS transgenic plants had lower levels of H_2_O_2_, the major indicator of ROS accumulation, and higher activities of antioxidant enzymes (SOD, CAT, POD, GR, and GPX) than WT plants under drought stress conditions (Fig. 2d–i). Previous studies in various plant species have shown that NO could modulate the activities of several antioxidant enzymes such as CAT, Fe-SOD, and dehydroascorbate reductase (DHAR) via *S*-nitrosylation modification (Shi and Chan, 2014). Based on the microarray data, although there were no significant changes in proline biosynthesis-related genes (Supplementary Table S1), many genes involved in redox metabolism (Supplementary Fig. S3a, b) and sugar metabolism (Supplementary Fig. S4a, b) were extensively changed by the nNOS transgene. As expected, some genes were up-regulated and others were down-regulated, indicating that NO had significant effects on the metabolism of these compounds partially through gene transcriptional modulation and protein post-translational modification. The results obtained with nNOS transgenic plants were consistent with those obtained with *arginase1/2* mutants, which exhibited increased *in vivo* NO content, enhanced activities of antioxidant enzymes, reduced water loss and EL, and thus increased stress tolerance relative to the WT (Shi *et al.*, 2013a). The reduced ROS accumulation and increased antioxidant enzyme activities confirmed that *in vivo* NO reduces drought stress-triggered oxidative stress and thereby reduces drought stress-triggered cell damage. These results, together with previously published observations (Gupta *et al.*, 2011; Shi et al., 2012*b*, *c*, 2013*a*; Tanou *et al.*, 2012; Shi and Chan, 2013), indicated that osmolyte accumulation, ROS accumulation, and antioxidant enzyme activities were important in drought stress tolerance. Additionally, Tanou *et al.* (2012) characterized some potentially carbonylated, nitrated, and nitrosylated proteins with distinct and overlapping signatures that belong to metabolic categories linked to ROS and NO acclimation signalling.

To gain insight into the NO-regulated defence response at the molecular level, comparative transcriptomic analysis was performed, and 490 and 20 genes that were differentially expressed in WT and nNOS transgenic plants under control and drought stress conditions, respectively, were identified (Fig. 3). Quantitative real-time PCR of 30 genes supported the reliability of the microarray analysis (Supplementary Fig. S2a–c at *JXB* online). Interestingly, far fewer genes were changed by drought stress in the WT (WT drought versus WT control, 154) than by the nNOS transgene under normal conditions (nNOS control versus WT control, 490). Based on physiological analyses, nNOS transgenic plants accumulated a high level of NO- and stress-related metabolites (proline, sugars, and antioxidants), which functioned as stress signals and therefore might affect a wide range of transcripts. Pathway enrichment analysis indicated that nine pathways were over-represented among differentially expressed genes in nNOS transgenic plants, including photosynthesis, hormone metabolism, redox, stress, mitochondrial electron transport, OPP, and lipid metabolism (Table 2). Additionally, 24 stress-related genes, and in particular 16 of them with log_2_ fold change ≥1, were significantly regulated by the nNOS transgene effect under control conditions (Supplementary Table S1). Among these stress-related genes, At5g66590 is involved in unspecified abiotic stress; At3g05890, At2g24040, and At2g45130 are involved in drought and salt stresses; At2g17880 and At4g36040 are involved in heat stress; and other genes are involved in biotic stress. All of these genes might contribute to the enhanced stress tolerance of nNOS transgenic plants, indicating that nNOS transgenic lines might be pre-conditioned to be resistant to abiotic stresses.

Previous studies have identified genes regulated by treatment with the NO donor SNP (Ahlfors *et al.*, 2009). Comparative analysis identified 22 genes that were regulated both by the nNOS transgene effect and by the SNP treatment, while six other genes showed the opposite expression pattern due to nNOS and SNP effects (Table 3). These 28 co-regulated genes might play essential roles in NO-mediated plant stress responses. A total of 165 genes that were regulated by both nNOS transgenic and ABA effects were also identified (Goda *et al.*, 2008), and most of them displayed the same pattern of up- and down-regulation by nNOS transgenic and ABA effects (Supplementary Table S4 at *JXB* online). The cross-talk between ABA and NO has been studied in depth, especially in terms of how it relates to abiotic stress tolerance (Guo *et al.*, 2003; Bright *et al.*, 2006; Lozano-Juste and León, 2010). Lozano-Juste and León (2010) found several ABA-related phenotypes as well as enhanced dehydration stress tolerance in NO-deficient plants, which might be due to the function of NO as an endogenous negative regulator of the sensitivity to ABA, thus leading to NO-independent inhibition of stomatal opening and enhanced closure by ABA. Among these 165 genes, the expression of two ABA receptor genes (*AtPYL4* and *AtPYL5*), which act upstream of the ABA pathway and directly modulate many downstream genes, was decreased by ABA and drought stress treatments (Chan, 2012), but increased in nNOS transgenic lines (Table 1; Supplementary Table S4), indicating a possible connection between NO and ABA pathways. Recent reports indicated that the constitutive overexpression of specific *PYL* genes in several plants enhanced their resistance to drought stress (Santiago *et al.*, 2009; Sun *et al.*, 2011). In this study, overexpression of *AtPYL4* and *AtPYL5* increased antioxidant enzyme activities and osmolyte levels and enhanced drought tolerance (Fig. 6; Supplementary Fig. S6). Up-regulation of *AtPYL4* and *AtPYL5* might contribute to the increased resistance of nNOS transgenic plants to drought stress. One possible mechanism might involve stomatal regulation because NO is involved in the stomatal closure triggered by ABA (Guo *et al.*, 2003). The increased NO content in nNOS transgenic plants (Supplementary Fig. S1b) might promote stomatal closure and result in the previously reported decrease in water loss (Shi *et al.*, 2012*c*). Additionally, the up-regulation of *AtPYL4* and *AtPYL5* by the endogenous NO content as a consequence of nNOS gene transformation or stress treatment might also contribute to improved drought stress resistance. Therefore, the co-regulation of genes by ABA and NO provides new clues regarding the interaction between ABA and NO signal transduction pathways.

Additionally, 14 members of the zinc finger family of proteins, a family that regulates the transcription of several stress-responsive genes (Miller *et al.*, 2008; Kodaira *et al.*, 2011; Liu *et al.*, 2012), were differentially expressed in WT versus nNOS transgenic plants (Supplementary Table S1 at *JXB* online). *AtZAT10* was commonly up-regulated by the nNOS transgenic effect and by SNP treatment (Table 3). The overexpression of zinc finger family protein genes such as *AZF1*, *AZF2*, *AZF3*, *ZAT6*, *ZAT7*, *ZAT10*, and *ZAT12* increased the tolerance of *Arabidopsis* to high light, high salt, drought, osmotic, and oxidative stresses (Miller *et al.*, 2008; Kodaira *et al.*, 2011; Liu *et al.*, 2012). Although the mechanisms by which different zinc finger proteins affect stress tolerance may be diverse and complex, the modulation of the expression of genes that encode several zinc finger family proteins by the nNOS transgene might contribute to the enhanced drought tolerance in nNOS transgenic plants. Moreover, the important role of some zinc finger proteins (AtZAT7, AtZAT10, and AtZAT12) in ROS signalling also suggests an important interaction among NO, zinc finger proteins, and ROS signalling in plant responses to stress. Additionally, *CBF/DREB* are known to increase the tolerance to abiotic stress (Seki *et al.*, 2001; Haake *et al.*, 2002; Sakuma *et al.*, 2006; Achard *et al.*, 2008; Novillo *et al.*, 2012), and their expression was greater in nNOS transgenic plants than in WT plants (Table 2; Supplementary Fig. S2a); it follows that the enhanced expression of *CBF1* and *CBF2* might also contribute to the enhanced stress tolerance of nNOS transgenic plants.

Taken together, the present comparative physiological and transcriptomic analyses revealed that WT and nNOS transgenic plants differed greatly in their physiological and molecular responses to drought stress. It is reasonable to infer that these differences might partially explain the difference in resistance to drought stress in WT versus nNOS transgenic plants. It is inferred that increased endogenous NO content resulting from nNOS transformation or from stress treatment modulates ROS accumulation, the activities of antioxidant enzymes, osmolyte levels, and the expression of stress-responsive genes (such as *AtPYL4* and *AtPYL5*), resulting in enhanced drought resistance. These finding increased our understanding of the physiological and molecular mechanisms by which NO mediates the drought stress response in *Arabidopsis*.

## Supplementary data

Supplementary data are available at at *JXB* online.

Table S1. Spreadsheet of all genes whose expression was changed by the nNOS transgene or drought stress treatment.

Table S2. Primers used for quantitative real-time PCR and vector construction.

Table S3. Gene lists of the 16 clusters analysed by MapMan software.

Table S4. Spreadsheet of genes commonly regulated by both the nNOS transgene effect and the ABA effect in *Arabidopsis*.

Figure S1. Improved drought stress resistance in nNOS transgenic *Arabidopsis* plants.

Figure S2. Verification of the microarray data by quantitative real-time PCR.

Figure S3. Metabolic pathway analyses of differentially expressed genes.

Figure S4. Effects of the nNOS transgene (a) and the drought stress treatment (b) on the plant photosynthesis pathway using MapMan software.

Figure S5. Enriched GO terms resulting from the nNOS transgene and the drought stress treatment.

Figure S6. Hierarchical cluster analysis of physiological parameters differentially expressed in WT, nNOS-, *AtPYL4*- and *AtPYL5*-overexpressing plants.

Supplementary Data
