# Supplementary material for: Constitutive production of nitric oxide leads to enhanced drought stress resistance and extensive transcriptional reprogramming in Arabidopsis
Source: J Exp Bot. 2014 May 27;65(15):4119–31. doi: 10.1093/jxb/eru184 (PMC4112625; doi:10.1093/jxb/eru184)
Supplement: Supplementary Data [file supp_65_15_4119__index.html]

Constitutive production of nitric oxide leads to enhanced drought stress resistance and extensive transcriptional reprogramming in Arabidopsis — Constitutive production of nitric oxide leads to enhanced drought stress resistance and extensive transcriptional reprogramming in Arabidopsis — Supplementary Data 

# Constitutive production of nitric oxide leads to enhanced drought stress resistance and extensive transcriptional reprogramming in *Arabidopsis*

## Supplementary Data

Data files

**Files in this Data Supplement:**

- Supplementary Data - Supplementary Data
- Supplementary Data - Supplementary Data
