# Supplementary material for: Constitutive production of nitric oxide leads to enhanced drought stress resistance and extensive transcriptional reprogramming in Arabidopsis
Source: J Exp Bot. 2014 May 27;65(15):4119–31. doi: 10.1093/jxb/eru184 (PMC4112625; doi:10.1093/jxb/eru184)
Supplement: Supplementary Data [file supp_eru184_jexbot117044_file001.pdf]

Supplementary data

Table S1. Spreadsheet of all genes whose expression was changed by the nNOS transgene or drought stress treatment.

Table S2. Primers used for quantitative real-time PCR and vector construction.

Table S3. Gene lists of the 16 clusters analyzed by MapMan software.

Table S4. Spreadsheet of genes commonly regulated by both the nNOS transgene effect and the ABA effect in *Arabidopsis*.

Fig. S1. Improved drought stress resistance in nNOS transgenic *Arabidopsis* plants. (a) Two-week-old plants were subjected to drought stress condition (or to well-watered, control condition) for 21 days before they were re-watered; the plants were photographed 7 days after watering was resumed; and the survival rate were also determined 7 days after watering was resumed. (b) NO content of WT and nNOS transgenic plants during control and drought stress conditions. The relative NO content of WT plants under control condition on 0 d was set at 1.0. Values are means ± SEs (n=4). Asterisks indicate significant differences between WT and nNOS transgenic plants (p<0.05).

Fig. S2. Verification of the microarray data by quantitative real-time PCR.

Fig. S3. Metabolic pathway analyses of differentially expressed genes. Metabolic pathways affected by the nNOS transgene (a) and by the drought stress treatment (b). Genes whose expression were differentially affected (p<0.05) were input and analyzed using MapMan software.

Fig. S4. Effects of the nNOS transgene (a) and the drought stress treatment (b) on the plant photosynthesis pathway using MapMan software.

Fig. S5. Enriched GO terms resulted from the nNOS transgene and the drought stress treatment.

Fig. S6. Hierarchical cluster analysis of physiological parameters differentially expressed in WT, nNOS, *AtPYL4* and *AtPYL5* overexpressing plants.

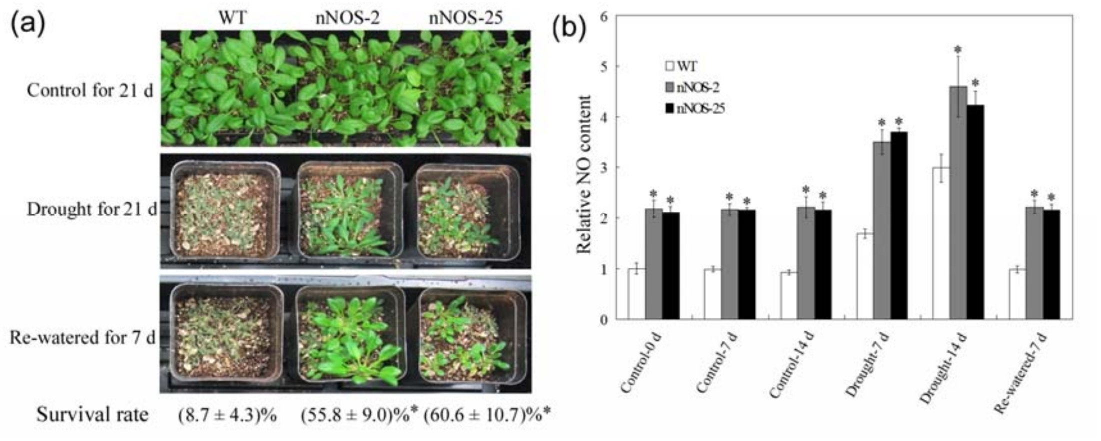
(a)
WT
nNOS-2
nNOS-25
Control for 21 d
Drought for 21 d
Re-watered for 7 d
Survival rate
(8.7 ± 4.3)%
(55.8 ± 9.0)%*
(60.6 ± 10.7)%*
(b)
WT
nNOS-2
nNOS-25
Relative NO content
0
1
2
3
4
5
6
Control-0 d
Control-7 d
Control-14 d
Drought-7 d
Drought-14 d
Re-watered-7 d

Fig. S1

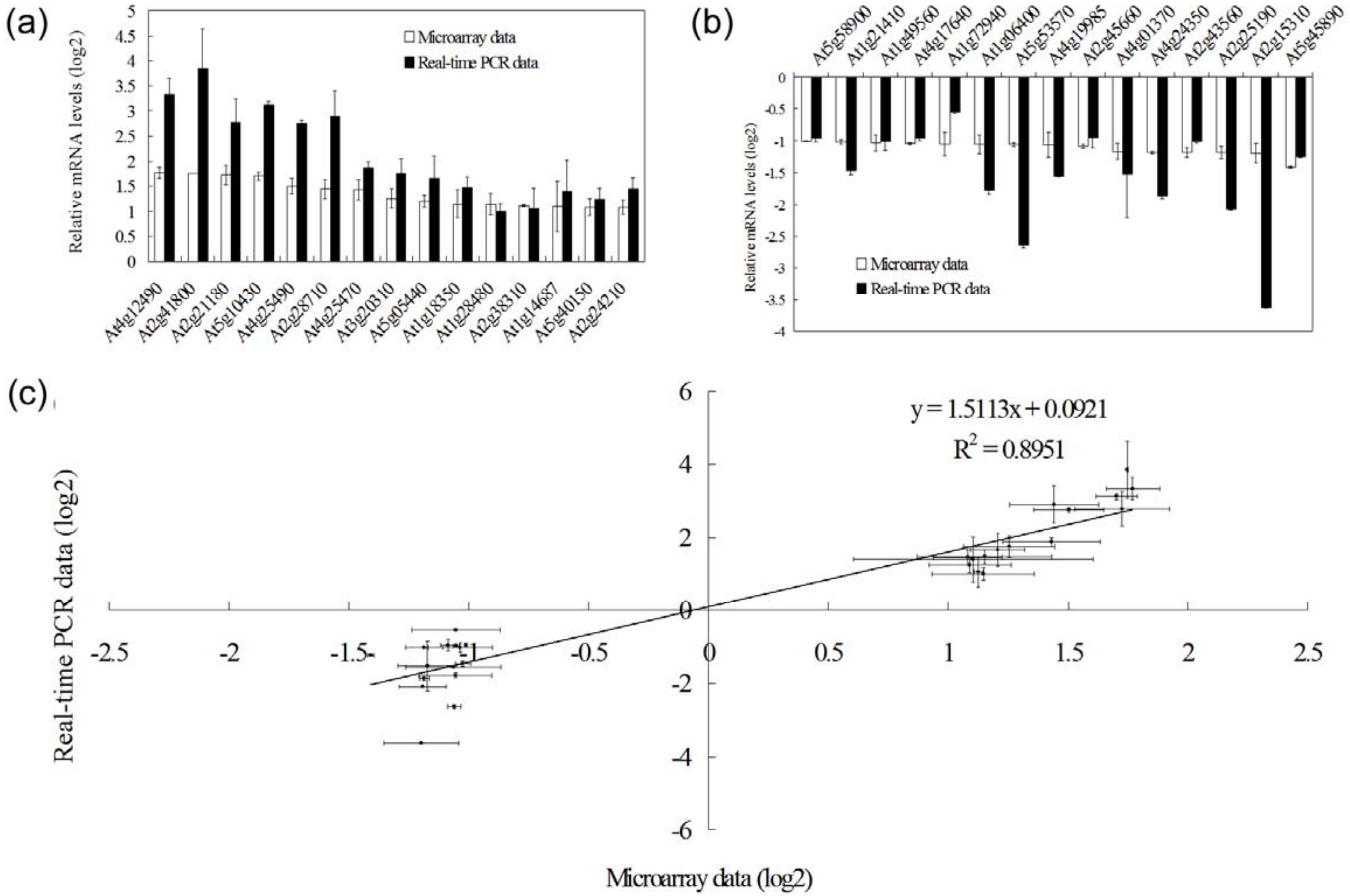
(a)
□ Microarray data
■ Real-time PCR data
Relative mRNA levels (log2)
At4g12490
At2g41800
At2g21180
At5g10430
At4g25490
At2g28710
At4g25470
At3g20310
At5g05440
At1g18350
At1g28480
At2g38310
At1g14687
At5g40150
At2g24210
(b)
At5g58900
At1g21410
At1g49560
At4g17640
At1g72940
At1g06400
At5g53570
At4g19985
At2g45660
At4g01370
At4g24350
At2g43560
At2g25190
At2g15310
At5g45890
Relative mRNA levels (log2)
□ Microarray data
■ Real-time PCR data
(c)
y = 1.5113x + 0.0921
R2 = 0.8951
Real-time PCR data (log2)
Microarray data (log2)

Fig. S2

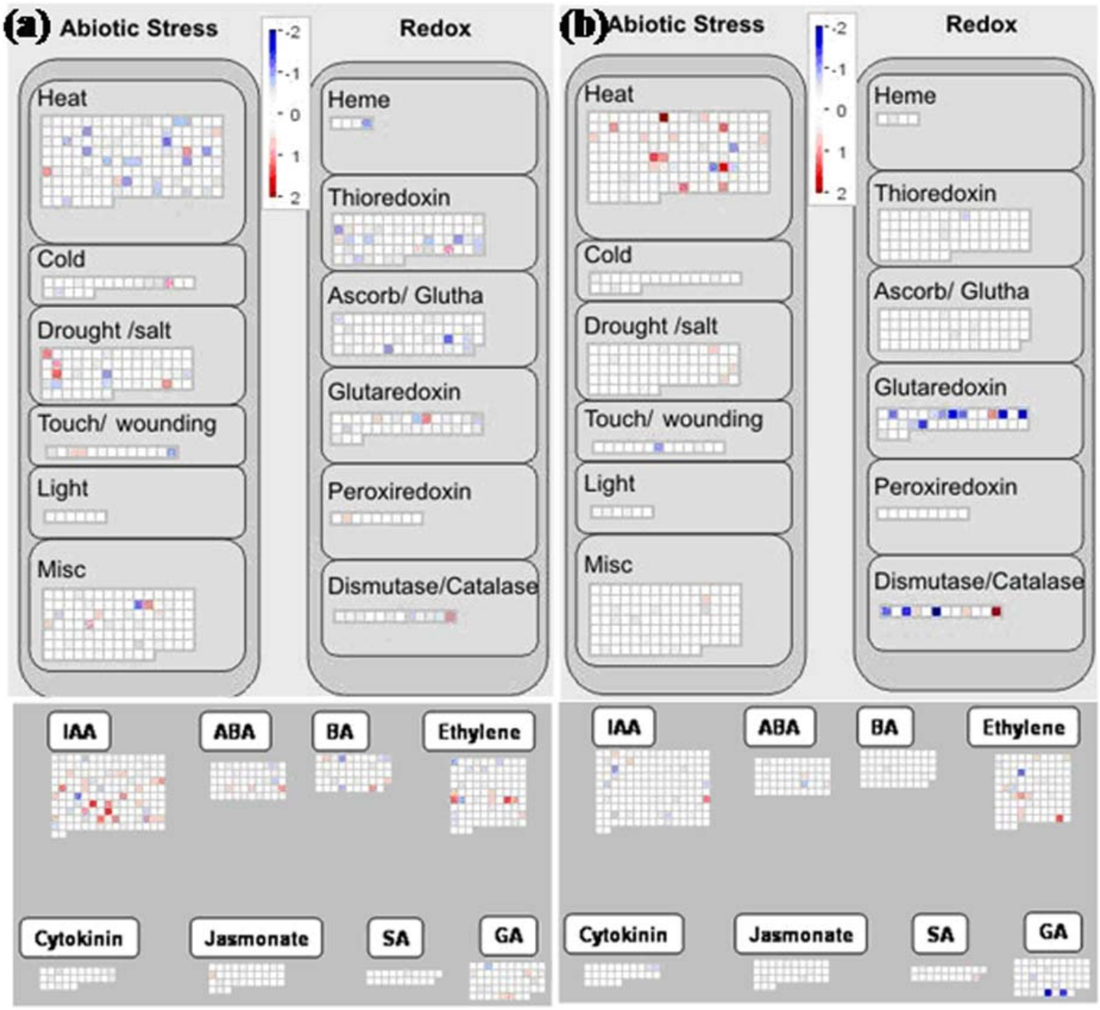
(a) Abiotic Stress
Redox
Heat
Cold
Drought /salt
Touch/ wounding
Light
Misc
Heme
Thioredoxin
Ascorb/ Glutha
Glutaredoxin
Peroxiredoxin
Dismutase/Catalase
-2
-1
0
1
2
IAA
ABA
BA
Ethylene
Cytokinin
Jasmonate
SA
GA
(b) Abiotic Stress
Redox
Heat
Cold
Drought /salt
Touch/ wounding
Light
Misc
Heme
Thioredoxin
Ascorb/ Glutha
Glutaredoxin
Peroxiredoxin
Dismutase/Catalase
-2
-1
0
1
2
IAA
ABA
BA
Ethylene
Cytokinin
Jasmonate
SA
GA

Fig. S3

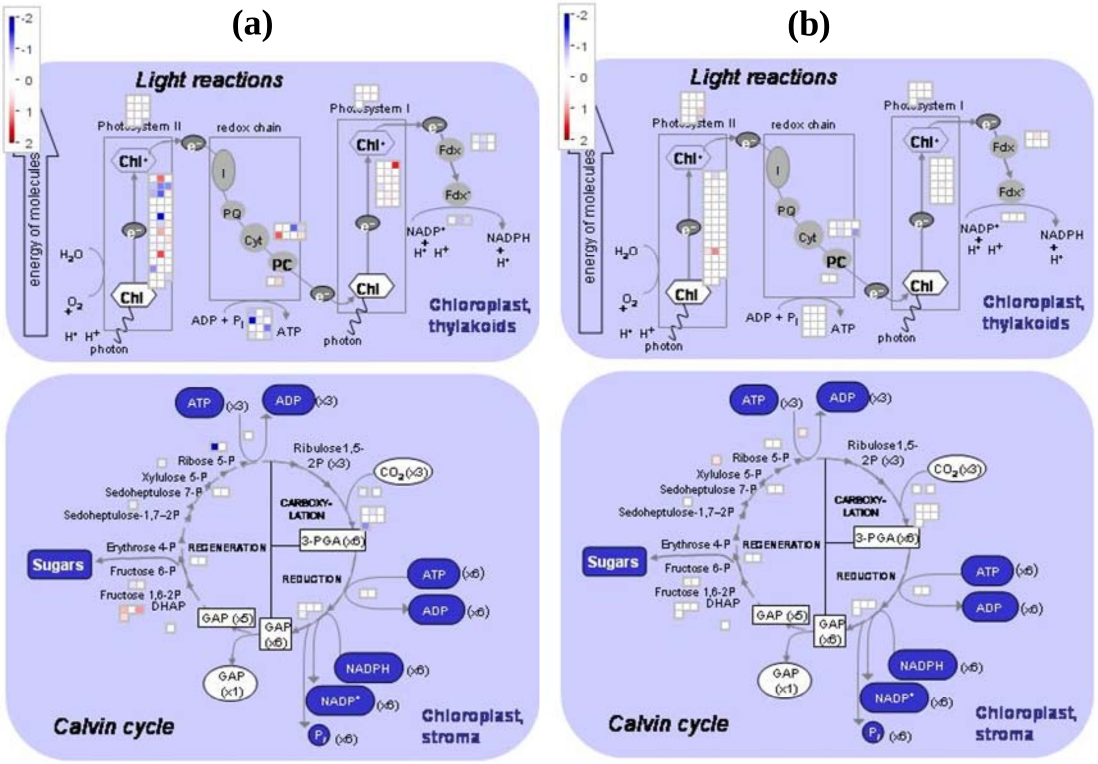
(a)
(b)
Light reactions
Photosystem II
redox chain
Photosystem I
energy of molecules
Chl*
Chl
PQ
Cyt
PC
Fdx
NADP+
NADPH
ADP + Pi
ATP
photon
Chloroplast, thylakoids
Calvin cycle
Chloroplast, stroma
Sugars
Ribose 5-P
Xylulose 5-P
Sedoheptulose 7-P
Sedoheptulose-1,7-2P
Erythrose 4-P
Fructose 6-P
Fructose 1,6-2P
DHAP
REGENERATION
CARBOXY-LATION
REDUCTION
3-PGA (x6)
GAP (x5)
GAP (x6)
GAP (x1)
Ribulose1,5-2P (x3)
CO2 (x3)

Fig. S4

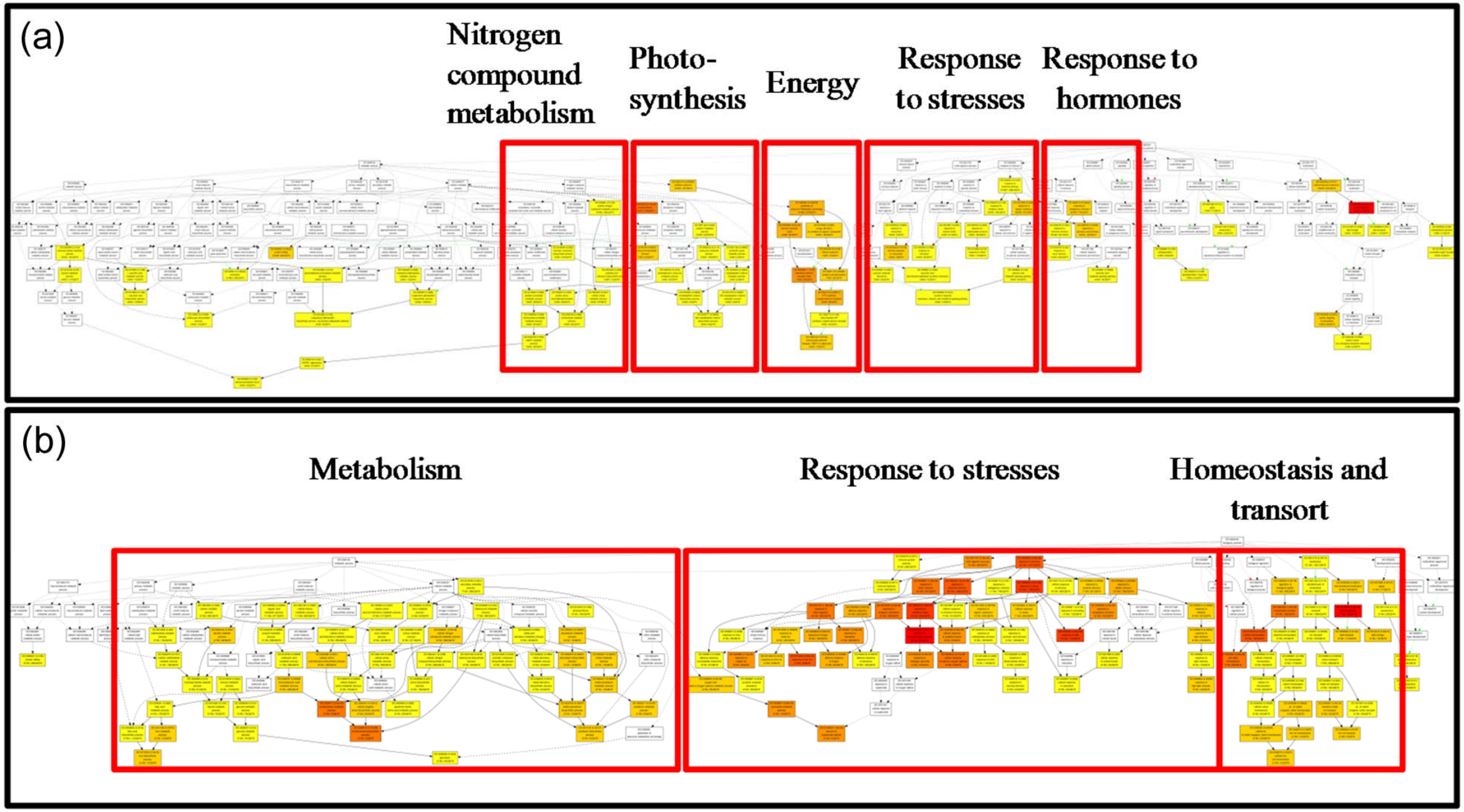
(a)
Nitrogen compound metabolism
Photo-synthesis
Energy
Response to stresses
Response to hormones
(b)
Metabolism
Response to stresses
Homeostasis and transort

Fig. S5

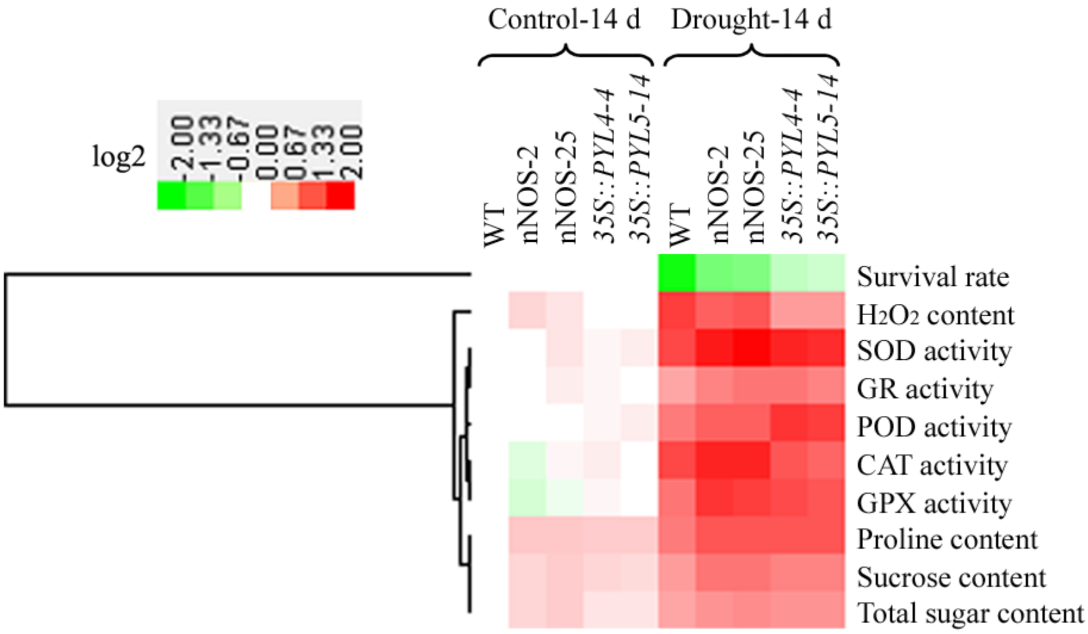
Control-14 d
Drought-14 d
log2
-2.00
-1.33
-0.67
0.00
0.67
1.33
2.00
WT
nNOS-2
nNOS-25
35S::PYL4-4
35S::PYL5-14
WT
nNOS-2
nNOS-25
35S::PYL4-4
35S::PYL5-14
Survival rate
H2O2 content
SOD activity
GR activity
POD activity
CAT activity
GPX activity
Proline content
Sucrose content
Total sugar content

Fig. S6
